# LncRNA SNHG3, a potential oncogene in human cancers

**DOI:** 10.1186/s12935-020-01608-x

**Published:** 2020-11-04

**Authors:** Bin Xu, Jie Mei, Wei Ji, Zheng Bian, Jiantong Jiao, Jun Sun, Junfei Shao

**Affiliations:** 1 Department of Neurosurgery, Wuxi People’s Hospital Affiliated to Nanjing Medical University, No. 299 Qing Yang Road, Wuxi, 214023 Jiangsu China; 2 Department of Oncology, Wuxi People’s Hospital Affiliated to Nanjing Medical University, Wuxi, P. R. China

**Keywords:** LncRNA, SNHG3, Cancer, Biomarker

## Abstract

Long noncoding RNAs (lncRNAs) are composed of > 200 nucleotides; they lack the ability to encode proteins but play important roles in a variety of human tumors. A large number of studies have shown that dysregulated expression of lncRNAs is related to tumor oncogenesis and progression. Emerging evidence shows that SNHG3 is a novel oncogenic lncRNA that is abnormally expressed in various tumors, including osteosarcoma, liver cancer, lung cancer, etc*.* SNHG3 primarily competes as a competitive endogenous RNA (ceRNA) that targets tumor suppressor microRNAs (miRNAs) and ceRNA mechanisms that regulate biological processes of tumors. In addition, abnormal expression of SNHG3 is significantly correlated with patient clinical features. Upregulation of SNHG3 contributes to biological functions, including tumor cell proliferation, migration, invasion and EMT. Therefore, SNHG3 may represent a potential diagnostic and prognostic biomarker, as well as a novel therapeutic target.

## Background

Recent studies have shown that cancer remains a global problem [1–3]. Numerous studies have shown that noncoding RNAs (ncRNAs) do not encode proteins but exert their function through various mechanisms, which are important aspects of cellular regulation [4–6]. Noncoding RNAs can be categorized according to length into small ncRNAs (< 200 nucleotides) and long ncRNAs (> 200 nucleotides, lncRNAs). LncRNAs are differentially classified based on place of their origin on the genome, including long intergenic noncoding RNA, natural antisense transcripts, circular RNA, pseudogene transcripts, transcribed ultraconserved regions, and telomerase RNA components [7–9].

LncRNAs are primarily transcribed by RNA polymerase II but are not translated into proteins [10, 11]. A growing number of studies have shown that lncRNAs play an important role in a variety of biological processes, including transcription, translation, and epigenetics. LncRNAs may act as tumor suppressor genes and oncogenes, affecting processes like cell proliferation, apoptosis, differentiation, invasion, migration, and suppression of immune responses. In addition, several lncRNAs exhibit cell- and tissue-specific expression patterns. Therefore, lncRNAs could be used as molecular markers for the early diagnosis of tumors and as novel targets for tumor treatment [12–18].

The small nuclear protein RNA host gene 3 (SNHG3) belongs to a group of long noncoding RNAs that are associated with multiple cancers and are dysregulated in multiple cancers. Recent studies have shown that SNHG3 expression is higher in many tumors compared to normal tissues. Furthermore, overexpression of SNHG3 significantly promotes tumor proliferation, migration and invasion, indicating that SNHG3 is a carcinogenic lncRNA. In addition, SNGH3 was previously identified by Honjo et al. as a nonimmunoglobulin target locus of the activation-induced cytidine deaminase (AID) pathway [19]. Lu et al. [20] found that SNHG3 is essential for mouse embryonic stem cell (mESC) self-renewal and pluripotency, as well as mouse early embryo development. This review summarizes research progress on the abnormal expression, functions, molecular mechanisms and clinical significance of SNHG3 in tumorigenesis and cancer progression (Tables 1, 2).

**Table 1:** Functional characterization of SNHG3 in various cancers

Cancer type	Expression	Role	Biological functions	Related genes	Refs.
Breast cancer	Up	Oncogenic	Promotes cell proliferation, migration and invasion	miR-384/HDGF	[31–33]
				miR-330-5p/PKM	
				miR-326/ITGA5, Vav2/Rac1	
Osteosarcoma	Up	Oncogenic	Promotes cell proliferation, migration, invasion and EMT	miR-151a-3p/RAB22A, miR-196a-5p/HOXC8	[25, 38]
Glioma	Up	Oncogenic	Promores cell proliferation, inhibits apoptosis, and regulates cell cycle	KLF2, p21, EZH2	[24]
Laryngeal cancer	UP	Oncogenic	Promotes cell migration, invasion and cell viability, glycolysis	miR-384/WEE1, MMP2, MMP9	[44, 45]
				miR-340-5p/YAP1, Wnt/β-catenin	
Gastric cancer	Up	Oncogenic	Promotes cell proliferation, migration, invasion and cell viability	EZH2, MED18	[23]
Colorectal cancer	Up	Oncogenic	Promotes cell proliferation, migration, invasion and cell viability	miR-182-5p/c-Myc, miR-539/RUNX2	[53, 54]
Renal cell carcinoma	Up	Oncogenic	Promotes cell proliferation, cell viability, migration, invasion and EMT and inhibits cell apoptosis	miR-139-5p/TOP2A, CDK6, Bax, Bcl-2, N-cadherin, E-cadherin, Vimentin	[57]
Hepatocellular carcinomac	Up	Oncogenic	Promotes cell proliferation, apoptosis, migration, invasion, EMT and sorafenib resistance	miR-139-5p/BMI1, miR-326/SMAD3/ZEB1, miR-128/CD151/AKT/PI3K, N-cadherin, E-cadherin, Vimentin, Snail	[60–63]
Lung cancer	Up	Oncogenic	Promotes cell proliferation, migration and EMT, inhibits cell apoptosis and regulates cell cycle	TGF-β, IL-6/JAK2/STAT3	[66, 67]
Acute myeloid leukemia	Up	Oncogenic	Promotes cell proliferation and inhibits cell apoptosis	miR-758-3p/SRGN	[70, 71]
Epithelial ovarian cancer	Up	Oncogenic	Promotes cell proliferation, invasion and glycolysis	CyclinD1, CDK1, MMP9, MMP3, GSK3β/β-catenin, EIF4A3-mRNA	[26, 74]
Papillary thyroid carcinoma	Up	Oncogenic	Promotes cell proliferation, migration and invasion	miR-214-3p/PSMD10	[76]
	Down	Tumor Suppressive	Promotes cell proliferation, migration and invasion	AKT/mTOR/ERK	[77]
Prostate cancer	UP	Oncogenic	Promotes cell proliferation, migration, invasion, EMT process and inhibits cell apoptosis	miR-577/SMURF1	[78]
Bladder cancer	Up	Oncogenic	Promotes cell proliferation, migration, invasion and EMT	miR-515-5p/GINS2	[79]
Oral squamous cell carcinoma	Up	Oncogenic	Promotes cell migration, invasion and proliferation	ELAVL1/NFYC, Wnt/β-catenin	[80]

*EMT* epithelial-mesenchymal transition, *HDGF* hepatoma-derived growth factor, *PKM* pyruvate kinase M1/M2, *ITGA5* integrin α5, *RAB22A* Ras-related proteins 22a, *HOXC8* homeobox c8, *KLF2* kruppel like factor 2, *p21* p21 protein, *EZH2* Enhancer of zeste homolog 2, *WEE1* wee1-like protein kinase, *MMP2* matrix metalloproteinase-2, *MMP9* matrix metalloproteinase-9, *YAP1* yes-associated protein 1, *MED18* mediator subunit18, *RUNX2* runt-related transcription factor 2, *TOP2A* the expression of topoisomerase IIα, *CDK6* cyclin-dependent kinase 6, *N-cadherin* neural-cadherin, *E-cadherin* epithelial-cadherin, *BMI1* B lymphoma Mo-MLV insertion region 1, *SMAD3* Sma and Mad Related Family 3, *ZEB1* zinc finger E-box binding homeobox 1, *PI3K* phosphoinositide-3 kinase, *TGF-β* transforming growth factor-β, *IL-6* the interleukin-6, *JAK2* Janus kinase 2, *STAT3* signal transducer and activator of transcription 3, *SRGN* serglycin, *GSK3β* glycogen synthase kinase 3 beta, *EIF4A3* eukaryotic translation initiation factor 4A3, *SMURF1* Smad Ubiquitin Regulatory Factor 1, *GINS2* GINS complex subunit 2, *PSMD10* proteasome 26S subunit non-ATPase 10, *ELAVL1* ELAV like RNA-binding protein 1, *NFYC* nuclear transcription factor Y subunit gamma

**Table 2:** Clinical significance of SNHG3 in diverse cancers

Cancer types	Overexpression of SNHG3 and clinical features	Refs.
Breast cancer	ER status, HER-2 status, tumor size, histological grade, lymph node metastasis and advanced TNM stage	[31–33]
Osteosarcoma	Tumor size and poor overall survival	[25, 38]
Glioma	Poor overall survival	[24]
Laryngeal cancer	No description	[44, 45]
Gastric cancer	Poor prognosis and lymph node metastasis	[23]
Colorectal cancer	Distant metastasis, advanced TNM stage and poor prognosis	[53, 54]
Renal cell carcinoma	Tumor size, distant metastasis, T stage, pathological TNM stage, histologic grade and poor prognosis	[57]
Hepatocellular carcinoma	Tumor size, histologic grade and poor prognosis	[60–63]
Lung cancer	Tumor size, TNM stage, lymph node migration and poor prognosis	[66, 67]
Leukemia	Poor prognosis	[70, 71]
Ovarian cancer	Poor prognosis, lymph node metastasis, FIGO stage and drug resistance	[26, 74]
Papillary thyroid carcinoma	Lymph node metastasis, tumor node metastasis stages and poor prognosis	[76, 77]
Prostate cancer	No description	[78]
Bladder cancer	Tumor size, metastasis and poor clinical prognosis	[79]
Oral squamous cell carcinoma	No description	[80]

*TNM* tumor node metastasis, *ER* estrogen receptor, *HER-2* human epidermal growth factor receptor-2, *FIGO* international federation of gynecology and obstetrics

## Characterization of SNHG3

**Fig. 1:**
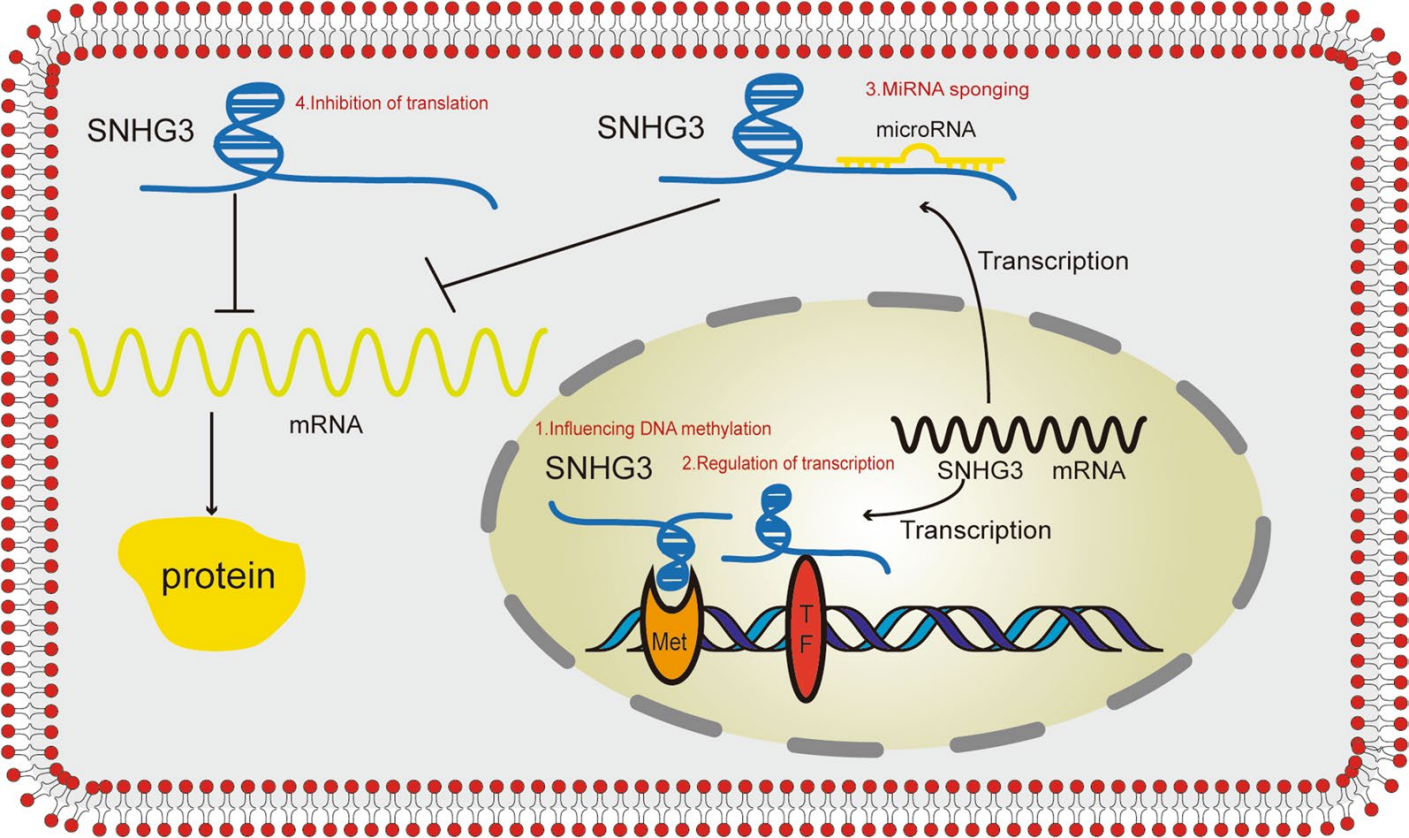
The regulatory molecular mechanisms of SNHG3 in tumorigenesis and progression. Nuclear: (1) DNA methylation is affected by the regulation of methylase. (2) Interaction with transcription factors and suppression of gene transcription. Cytoplasm: (3) MiRNA sponging and miRNA target release. (4) Inhibition of translation

Small nucleolar RNAs (snoRNAs) are primarily transcribed from protein coding gene clusters or other ncRNA coding genes. Some snoRNA genes without coding ability still contain introns and exons in their sequences, but snoRNAs are only produced by introns. If full-length transcripts, including exons, are retained, they will function as lncRNAs, called small nucleolar RNA host genes (SNHGs) [21]. SNHG3 is an oncogenic lncRNA that produces SNORD17. SNHG3 is not only found in the nucleus but also in the cytoplasm [22] (Fig. 1). SNHG3 is located on chromosome 1p35.3 and has four exons that comprise 4950 bp. The Human Protein Atlas (HPA) project RNA-seq normal tissue results suggest that SNHG3 is more highly expressed in the bone marrow and appendix than in other human tissues. In the SNHG3 literature, we identified four major molecular mechanisms of action with different subcellular localizations. Nuclear: (1) DNA methylation is affected by the regulation of methylase. For instance, SNHG3 promotes gastric cancer progression through regulating neighboring mediator subunit 18 (MED18) gene methylation by binding to enhancer of zeste homolog 2 (EZH2) [23]. (2) Interaction with transcription factors and suppression of gene transcription. For instance, SNHG3 enhances the malignant behaviors of glioma through silencing Kruppel-like factor 2 (KLF2) and p21 protein (p21) via recruiting EZH2 to the promoter of KLF2 and p21 [24]. Cytoplasm: (3) MiRNA sponging and miRNA target release. For instance, SNHG3 sponges miR-151a-3p as a ceRNA to upregulate Ras-related protein 22a (Rab22a) expression, thus increasing cell migration and invasion of osteosarcoma cells [25]. (4) Inhibition of translation. For instance, SNHG3 is related to energy metabolism by regulating eukaryotic translation initiation factor 4A3 (EIF4A3) mRNA in ovarian cancer (OC) [26]. A growing number of studies have shown that SNHG3 plays a crucial role in the development and prognosis of a variety of malignancies.SNHG3 may represent a valuable prognostic biomarker and therapeutic target in various cancers.

## LncRNA SNHG3 dysregulation in human cancers

### LncRNA SNHG3 in breast cancer

Breast cancer is the most common cancer among American women and the second leading cause of cancer death in women, second only to lung cancer [27, 28]. Metastasis of cancer cells is one of the leading causes of death in breast cancer patients [29]. Although significant progress has been made in the diagnosis and treatment of breast cancer, the mortality rate of breast cancer patients remains high [30]. Therefore, further study of the molecular mechanism of breast cancer is crucial. Ma et al. [31] examined SNHG3 expression in 60 pairs of breast cancer tissues and observed remarkable upregulation of SNHG3 expression in breast cancer tissues and cells. In addition, its overexpression was significantly associated with histological grade, lymph node metastasis, advanced tumor node metastasis (TNM) stage, and estrogen receptor (ER) and human epidermal growth factor receptor 2 (Her-2) status. Functionally, SNHG3 knockdown hindered breast cancer cell proliferation, migration and invasion in vitro and in vivo. Moreover, SNHG3 expression is positively correlated with hepatoma-derived growth factor (HDGF) expression. Furthermore, SNHG3 acts as a competing endogenous RNA by directly binding to miR-384 in a sequence-specific manner. Meanwhile, HDGF is also a target gene of miR-384 and is regulated by SNHG3. In other words, SNHG3 functions as an endogenous sponge by sequestering miR-384, thus abolishing the miRNA-induced repressive effect on HDGF. Yan et al. [32] reported that SNHG3 uses a miR-330-5p sponge to positively regulate pyruvate kinase M1/M2 (PKM) expression, inhibit mitochondrial oxidative phosphorylation, and enhance breast cancer cell proliferation. Wang et al. [33] found that SNHG3 silencing enhances apoptosis in triple-negative breast cancer (TNBC) cells through the miR-326/integrin α5 (ITGA5) axis and inhibits cell viability, migration, invasion and the Vav2/Rac1 signaling pathway. These results demonstrated that SNHG3 acts as an oncogenic lncRNA in breast cancer, which may represent a potential diagnostic biomarker or a novel therapeutic target for cancers [34].

### LncRNA SNHG3 in osteosarcoma

Osteosarcoma (OS) is the most common malignant bone tumor, primarily affecting adolescents. OS frequently affects long bones and is prone to lung metastasis [35]. Although treatment of OS has made great progress, the prognosis of OS patients is still poor due to distant metastasis [36]. Therefore, novel and effective prognostic biomarkers and therapeutic targets for the disease must be identified [37]. Zheng et al. [25] found that expression of SNHG3 and Rab22a was upregulated in 54 OS tissues and OS cell lines (Saos2, MG63, U2OS and HOS) compared to paired normal tissues and normal osteoblasts, while miRNA-151a-3p was expressed at low levels. Overexpression of SNHG3 accelerates the invasive and migratory potentials of OS cells. SNHG3 overexpression in MG63 cells markedly enhanced their phenotype, and SNHG3 knockdown in U2OS cells inhibited their phenotype. SNHG3 upregulates Rab22a expression, and SNHG3 binds to Rab22a and miRNA-151a-3p. A previous study demonstrated that the SNHG3/miRNA-151a-3p/Rab22a axis regulates invasion and migration of osteosarcoma. Chen et al. [38] also confirmed that expression of SNHG3 was upregulated in both OS tissues and OS cell lines compared to paired normal tissues and normal osteoblasts. Overexpression of SNHG3 markedly increases cell viability and colony number. SNHG3 acts as a sponge of miRNA-196a-5p in OS cells. These results indicated that SNHG3 promotes the growth of OS cells by sponging the miRNA-196a-5p/homeobox c8 (HOXC8) axis, providing a potential marker in OS patients.

### LncRNA SNHG3 in glioma

Glioma is one of the most common major brain tumors in adults and one of the most important malignant tumors in humans [1]. Although surgery, chemotherapy, radiotherapy and other treatments have made great progress, the clinical prognosis of glioma patients remains poor [39]. Therefore, it is important to understand the molecular mechanisms underlying the development of gliomas [40]. Fei et al. [24] uncovered high expression levels of SNHG3 in glioma tissues and glioma cell lines compared to nontumor tissues and normal cells. Furthermore, upregulated SNHG3 suggests a poor prognosis in patients with glioma. Silencing of SNHG3 inhibits cell proliferation by triggering apoptosis and cell cycle arrest. After SNHG3 was overexpressed in U251 cells, the opposite results were observed. In addition, KLF2 and p21 were downregulated in glioma tissues. SNHG3 was negatively correlated with p21 and KLF2. These experiments revealed that SNHG3 accelerates the malignancy of glioma by inhibiting transcription of KLF2 and p21. Generally, it is believed that SNHG3 may be an oncogene in glioma and may serve as a potential prognostic biomarker and therapeutic target for glioma.

### LncRNA SNHG3 in laryngeal cancer

Laryngeal cancer (LC) is a common malignant tumor of the head and neck, whose main pathological type is squamous cell carcinoma. The primary risk factors for LC include smoking, alcohol consumption, environmental factors, radioactivity, viral infections, and trace element deficiency [41]. Current treatment strategies include radiation therapy and surgical removal of tumors [42]. Therefore, early diagnosis and treatment are essential for maintaining laryngeal function and reducing postoperative complications [43]. Wang et al. [44] determined that expression of SNHG3 was upregulated in LC tissues and cell lines compared to normal tissues and cell lines. Moreover, downregulation of SNHG3 significantly depressed migration and invasion capacities, as well as matrix metalloproteinase-2 (MMP2) and matrix metalloproteinase-9 (MMP9) protein levels. SNHG3 negatively regulates miR-384, improves wee1-like protein kinase (WEE1) expression, and promotes LC cell migration and invasion. Furthermore, SNHG3 acts as a ceRNA by directly binding to miR-384 to increase WEE1 expression. In summary, these results indicated that SNHG3 regulates the migration and invasion of LC cells via the miR-384/WEE1 axis. Kang et al. [45] found that knock down of SNHG3 reduced the growth of LSCC xenograft tumors by regulating the miR-340-5p/yes-associated protein 1 (YAP1) axis and Wnt/β-catenin pathway. As mentioned above, SNHG3 may represent a potential therapeutic target for LC patients.

### LncRNA SNHG3 in gastric cancer

Gastric cancer (GC) is one of the most common malignancies in humans [46]. The most common cause of gastric cancer is *Helicobacter pylori* (*Hp*) infection, accounting for more than half of the total incidence [47, 48]. GC treatment primarily includes surgery, chemotherapy, radiotherapy and targeted therapy [49]. Early diagnosis helps patients who are in the early stages of disease progression. There is ample evidence that lncRNAs play an indispensable role in promoting tumor or antitumor effects in human malignancies [50]. Xuan et al. [23] observed apparent overexpression of SNHG3 in cancer cell lines and tissues in comparison with normal cells and tissues. High SNHG3 in GC also predicted poor prognosis. SNHG3 promoted the proliferation of gastric cancer cells both in vitro and in vivo. Conversely, downregulation of SNHG3 inhibited the migration, invasion and metastasis of GC cells both in vitro and in vivo. SNHG3 and EZH2 simultaneously bind to MED18 promoter, and SNHG3 exerts apparent regulation of neighboring gene MED18 transcription in GC cells. In conclusion, their results demonstrated that MED18 exhibits antitumor activity in GC cells by inhibiting cell proliferation, migration and invasion. The anticancer effect of MED18 occurs downstream of SNHG3 signaling in gastric cancer, which may imply its widespread mode of action in other cancers, and deserves further study.

### LncRNA SNHG3 in colorectal cancer

Colorectal cancer (CRC) is the third most common malignancy in the world with high morbidity and mortality rates [46]. Although advanced treatments exist, including surgical resection, chemotherapy, radiation, the 5-year and 10-year survival rates in CRC patients are still unsatisfactory at 65% and 58%, respectively [51]. In summary, a better understanding of the precise molecular mechanisms that promote the development of CRC will be beneficial to help CRC patients and to identify new diagnostic methods or treatment strategies [52]. Huang et al. [53] showed that SNHG3 was highly expressed in CRC tumor tissues compared to adjacent normal tissues. Overexpression of SNHG3 significantly increased the proliferative ability of CRC cells. Consistently, SNHG3 downregulation inhibited CRC cell proliferation. Their results indicate that SNHG3 and c-Myc share the same miRNA-responsive element with miR-182-5p and promote CRC progression in a ceRNA manner. Wen et al. [54] revealed that knockdown of the SNHG3 gene significantly reduces growth and metastasis of CRC. Mechanistically, SNHG3 acts as the miRNA's ceRNA to bind miR-539, thereby regulating the expression of its target gene, runt-related transcription factor 2 (RUNX2), and promoting the occurrence and development of CRC. Upregulation of SNHG3 is correlated with poor prognosis, indicating that it may be an important biomarker of CRC.

### LncRNA SNHG3 in renal cell carcinoma

Renal cell carcinoma (RCC) accounts for 5% of adult malignancies. It is estimated that there were approximately 73,820 new cases and 14,770 renal cell carcinoma deaths in the United States in 2019 [46]. Conventional radiotherapy and chemotherapy are not ideal, and surgery is the primary method for treating renal cell carcinoma. In recent years, targeted therapy has been found to improve patient prognosis, but the emergence of drug resistance has hindered the development of targeted drugs. Therefore, it is crucial to further identify new therapeutic targets for RCC [55, 56]. Zhang et al. [57] found that SNHG3 acts as a ceRNA of mir-139-5p and inhibits the activity of mir-139-5p. Expression of topoisomerase IIα (TOP2A), a target of miR-139-5p, was increased, which ultimately promoted proliferation and metastasis of renal cancer cells. These results confirmed that the SNHG3/miR-139-5p/TOP2A axis plays a crucial role in renal cell carcinoma and may represent a key target for diagnosis and treatment.

### LncRNA SNHG3 in hepatocellular carcinoma

Hepatocellular carcinoma (HCC) is one of the major malignancies in the world [58]. Over the years, there have been several advances in the diagnosis and treatment of HCC. Due to its high recurrence rate and high distant metastasis rate, the overall survival (OS) and 5-year survival rate of HCC patients are still low. Therefore, it is very urgent to further study the pathogenesis of HCC and discover new targets for diagnosis and treatment [59]. Wu et al. [60] reported that the lncSNHG3/miR-139-5p/B lymphoma Mo-MLV insertion region 1 (BMI1) axis plays an important role in cell proliferation, migration, and invasion in HCC. Zhao et al. [61] showed that overexpression of SNHG3 promotes proliferation, migration and epithelial-mesenchymal transition (EMT) of HCC and inhibits apoptosis, while knockdown of SNHG3 plays the opposite role. The proposed mechanism suggested that SNHG3 may act as a ceRNA of miR-326, increasing expression levels of Sma and mad related family 3 (SMAD3) and zinc finger E-box binding homeobox 1 (ZEB1). In conclusion, SNHG3 promotes hepatocellular tumorigenesis via the miR-326/SMAD3/ZEB1 axis. Zhang et al. [62] found that expression of SNHG3 in highly metastatic HCC cells was significantly higher than in low metastatic HCC cells. Functionally, high expression of SNHG3 promotes cell invasion, EMT and sorafenib resistance. In addition, SNHG3 acts as a ceRNA and induces EMT in hepatoma cells via the miR128/CD151 signaling pathway. Zhang et al. [63] reported that elevated expression of SNHG3 in liver cancer patients was associated with malignant status and poor prognosis. Taken together, their data suggest that SNHG3 may represent a new therapeutic target and a biomarker for predicting HCC response to sorafenib [64].

### LncRNA SNHG3 in lung cancer

Lung cancer is a major, worldwide, malignant and deadly tumor. Clinically, methods for treating lung cancer include radiotherapy, chemotherapy, surgery, etc. However, lung cancer patients have poor prognosis, and the 5-year survival rate is only 16% [1, 46]. To further elucidate the molecular mechanism of lung adenocarcinoma, we are looking for new diagnostic and therapeutic targets for the treatment of lung adenocarcinoma [65]. Shi et al. showed that expression of SNHG3 in non-small-cell lung cancer (NSCLC) tissues and cells was higher than in normal tissues and cell lines. In addition, NSCLC patients with high SNHG3 expression had a low overall survival rate. Functionally, high expression of SNHG3 significantly promotes cell proliferation and migration. Mechanistically, SNHG3 is activated by E2F transcription factor 1 (E2F1), subsequently promoting proliferation and migration of NSCLC by activating the transforming growth factor‐β (TGF-β), interleukin‐6 (IL-6)/janus‐activated kinase 2 (JAK2)/signal transducer activator of transcription 3 (STAT3) pathways [66]. Liang et al. [67] also confirmed that overexpression of SNHG3 promotes lung adenocarcinoma cell proliferation and the cell cycle, as well as inhibiting apoptosis. Therefore, SNHG3 may be a potential new target for the treatment and prognosis of lung adenocarcinoma.

### LncRNA SNHG3 in leukemia

Leukemia is a relatively rare cancer with a median age of approximately 60 years [68]. A considerable number of studies have shown that acute myeloid leukemia (AML) is a highly heterogeneous collection of diseases that may be genetically altered in terms of cell morphology, cytochemistry, immunophenotype, cytogenetics, and molecular abnormalities [69]. Therefore, it is urgent to further study the mechanism of leukemia occurrence and development to identify new biomarkers and treatment strategies. Peng et al. [70] revealed that silencing SNHG3 inhibits cell proliferation and induces apoptosis. In addition, downregulation of SNHG3 significantly reduced the expression of SRGN in AML cells. Mechanistically, we found that SNHG3 regulates the expression of serglycin (SRGN) by competitively binding to miR-758-3p. In other words, SNHG3 promotes the growth of acute myeloid leukemia cells by regulating the miR-758-3p/SRGN axis, providing a new therapeutic direction for the treatment of AML. According to the dynamic network biomarker (DNB) standard, some dysregulated lncRNA-associated ceRNA network biomarkers for chronic myeloid leukemia (CML) have been identified and analyzed. Xu et al. [71] found that SNHG3 is an effective CML biomarker that helps patients obtain timely treatment and reduces CML mortality.

### LncRNA SNHG3 in epithelial ovarian cancer

Epithelial ovarian cancer (EOC) is the deadliest gynecological malignancy. Due to the asymptomatic and concealed nature of ovarian cancer, EOC patients are often clinically advanced when ultimately diagnosed, which poses a huge obstacle to clinical diagnosis and treatment [72]. Therefore, it is urgent to further elucidate the molecular mechanism of ovarian cancer to identify molecules for early diagnosis. Li et al. [73] found that lncRNA SNHG3 regulates energy metabolism in ovarian cancer as shown by mitochondrial proteomic analysis. Hong et al. [74] showed that expression of SNHG3 in ovarian cancer tissues was significantly higher than in adjacent normal tissues. In addition, SNHG3 was highly positively associated with FIGO stage, lymph node metastasis, and poor prognosis. Overexpression of SNHG3 promoted the proliferation and invasion of ovarian cancer cells, resulting in a significant downregulation of CyclinD1, CDK1, MMP9 and MMP3. Results also demonstrated that regulation of the glycogen synthase kinase 3 beta (GSK3β)/β-catenin signaling pathway promotes the development of ovarian cancer. Li et al. [26] uncovered that ivermectin inhibits OC migration by potentially targeting the lncRNA-EIF4A3-mRNA pathway, establishing an effective prognostic model. These findings indicate that OC treatment and parents' postoperative pain measurement (PPPM) prognostic assessment have practical significance. In conclusion, SNHG3 may serve as a novel target for the diagnosis and treatment of ovarian cancer.

### LncRNA SNHG3 in papillary thyroid carcinoma

Papillary thyroid cancer (PTC) is the most common type of thyroid cancer and ranks ninth in global cancer incidence [46]. Although most PTCs show good prognosis, a small number of patients experience aggressive and refractory disease [75]. Therefore, it is urgent to understand the molecular mechanism of PTC tumor occurrence and progression to further improve the cure rate and survival rate in patients. Sui et al. [76] showed that SNHG3 can be used as the ceRNA of this miRNA to bind to miR-214-3p, actively regulating expression of the proteasome 26S subunit non-ATPase 10 (PSMD10) and significantly reducing PTC in vitro cell migration, invasion, proliferation and colony formation. These results suggest that SNHG3 may be a diagnostic and therapeutic target for PTC. Duan et al. [77] found that lncRNA SNHG3 was significantly downregulated in PTC tissues and cell lines. Functional studies have shown that SNHG3 deletion promotes the proliferation, migration and invasion of PTC cells. Further mechanistic analysis showed that knockout of SNHG3 promotes the occurrence of PTC tumors both in vivo and in vitro by activating the AKT/mTOR/ERK pathway. Of note, SNHG3 may become a promising candidate for PTC targeted therapy.

### SNHG3 in other cancers

Li et al. [78] reported that SNHG3 is highly expressed in prostate cancer cell lines. SNHG3 knockdown inhibits prostate cancer cell proliferation, migration, and EMT processes, promoting cell growth. Mechanistically, SNHG3 endogenously absorbs miR-577, thereby positively expressing smad ubiquitin regulatory factor 1 (SMURF1) and promoting the development of prostate cancer. We experimentally discovered demonstrated that the carcinogenic effects of SNHG3 and SMURF1 in prostate cancer may provide new ideas for biomarkers of prostate cancer. Dai et al. [79] identified that lncRNA SNHG3 is upregulated in bladder cancer tissues, and lncRNA SNHG3 knockdown inhibits bladder cancer cell proliferation, migration, invasion and EMT processes both in vitro and in vivo. In addition, under the mechanism of ceRNA, SNHG3 sponges miR515-5p to escalate expression of the GINS complex subunit 2 (GINS2). Therefore, research shows that lncRNA SNHG3 may become a new diagnostic and treatment target for bladder cancer. Liu et al. [80] confirmed that SNHG3 participates in the progression of oral squamous cell carcinoma (OSCC) by regulating the ELAV-like RNA-binding protein 1 (ELAVL1)/nuclear transcription factor Y subunit gamma (NFYC) axis and the Wnt/β-catenin pathway. In summary, SNHG3 may represent a new biomarker for OSCC.

## Perspectives in clinical practice

### Diagnostic and prognostic value of SNHG3 in cancer assessment

Pancancer analysis reveals that long-chain noncoding RNAs have potential diagnostic and prognostic value in a variety of cancers [81]. With the continuous development of RNA sequencing, gene microarrays and high-throughput sequencing, we recognize that lncRNAs can act as tumor suppressors and oncogenes, playing key roles in regulating tumor formation and development [82]. With the study of the molecular mechanism of lncRNAs in tumors, lncRNAs may become new tumor biomarkers for the diagnosis and treatment of cancer targets [83]. Yang et al. [84] used RNA-seq and survival data in the TCGA database to analyze the expression profiles of 20 SNHGs and discussed their prognostic value in clear cell renal cell carcinoma (ccRCC). Results of the study indicated that SNHG3 and SNHG15 act as diagnostic markers and indicators and may have great clinical value to evaluate the survival and progression of ccRCC. Although there are still quite a few challenges remaining to be solved, the role of lncRNAs in clinical practice is a new hot spot. Multiple scholars have tried to use SNHG3 as a biomarker and therapeutic target for the diagnosis, treatment and monitoring of tumors. As mentioned earlier, many studies have shown that compared to normal tissues, tumor tissues have increased abnormally expressed SNHG3, which may be used as potential biomarkers for tumor diagnosis.

### SNHG3 as a potential therapeutic tool in cancer treatment

It has been proven that abnormal small ncRNA expression levels in tumor cells can be used as effective drug targets for the treatment of tumors [85]. A wide range of ncRNAs have been discovered, namely, microRNAs (miRNAs), small interfering RNAs (siRNAs), and piwi-interacting RNAs (piRNAs). After these small RNAs (sRNAs) are incorporated into miRNA or siRNA-induced silencing complex (RISC), they participate in gene silencing in cells primarily through RNAi interference (RNAi) mechanisms [86, 87]. What’s more, antisense oligonucleotides (ASOs) are short single-stranded nucleic acid sequences, which form a stable hybrid with its target mRNA, thereby interfering with its processing or translation [88]. Although small ncRNAs have proven to be promising in vitro effective therapeutic drugs, it is very difficult to deliver these nucleic acid drugs into cells, and the low bioavailability of these nucleic acid drugs in vivo remains a major challenge. Therefore, ncRNA needs to be transported to the target tissue by a suitable carrier. Various small ncRNA vectors or systems have been proposed and widely explored for this purpose, including nanoparticles, ncRNA modification, and oncolytic adenovirus strategies. Nanoparticle-based small ncRNA vectors are the most common strategy [89]. SNHG3 is located in the nucleus and cytoplasm and requires a carrier to transport antisense RNAs or siRNAs into the cell to function [90]. Therefore, through continuous progress of antisense RNA or siRNA and ncRNA vectors, direct silencing of SNHG3 has become an important and powerful new target for tumor treatment.

## Conclusions and future perspectives

**Fig. 2:**
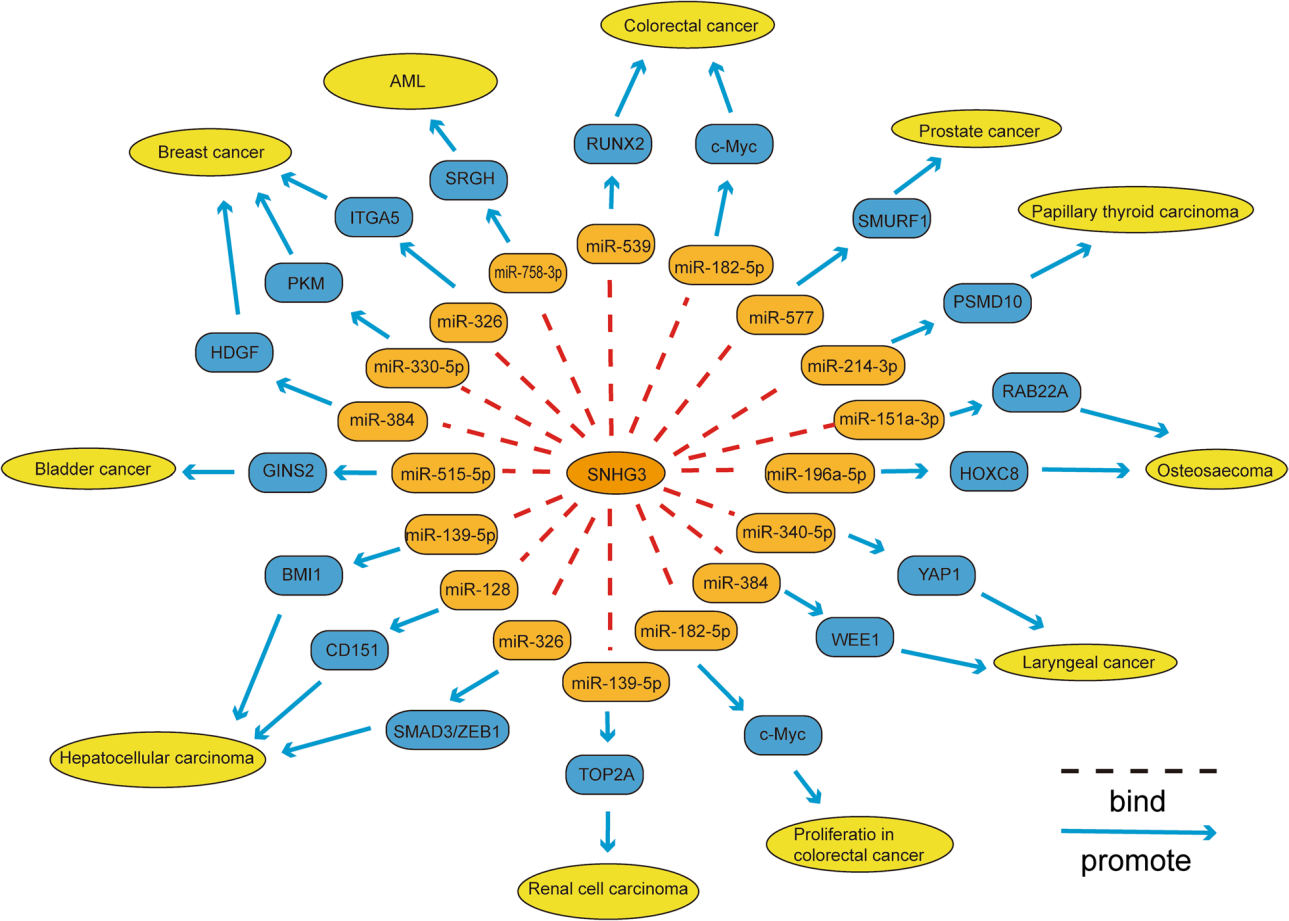
SNHG3 as a competing endogenous RNA (ceRNA) in a variety of cancers. SNHG3 regulates the expression of mRNAs by competitively binding microRNAs, such as miR-384, miR-330-5p, miR-326, miR-151a-3p, miR-196a-3p, miR-384, miR-340-5p, miR-182-5p, miR-539, miR-139-5p, miR-326, miR-128, miR-758-3p, miR-577, miR-515-5p, and miR-214-3p, which control cell proliferation, apoptosis, migration, invasion, EMT, cell cycle arrest, cell viability and therapeutic resistance

Many studies have shown that lncRNA SNHG3 is overexpressed in most tumors, including breast cancer, osteosarcoma, glioma, laryngeal cancer, gastric cancer, colorectal cancer, renal cell carcinoma, hepatocellular carcinoma, lung cancer, acute myeloid leukemia, and epithelial ovarian cancer. Aberrant expression of SNHG3 is significantly associated with clinical features, such as tumor size, TNM stage, lymph node metastasis, and overall patient survival. At the same time, abnormal expression of SNHG3 plays an important role in tumor biology in processes such as proliferation, migration, invasion and attenuation of apoptosis in tumor cells. In addition, a growing number of studies have shown that SNHG3, a ceRNA, competes with endogenous miRNA for binding, thereby inhibiting downstream genes of these miRNAs or regulating the development of tumor cells through classical pathways (Fig. 2). Therefore, SNHG3 will likely serve as a new target for tumor diagnosis and as a prognostic biomarker or a target for treating tumors. Although the molecular mechanisms of SNHG3 in many tumors have been studied, many questions remain. In addition, the precise molecular mechanism of SNHG3 requires further study, which will help in applying the results of SNHG3 research to clinical diagnosis and treatment of cancer.

## Data Availability

All data are included in the article.

## Abbreviations

lncRNAs: Long noncoding RNAs
ceRNA: Competitive endogenous RNA
miRNAs: MicroRNAs
ncRNA: Noncoding RNAs
SNHG3: The small nuclear protein RNA host gene 3
AID: Activation-induced cytidine deaminase
mESC: Mouse embryonic stem cell
SnoRNAs: Small nucleolar RNAs
SNHG: Small nucleolar RNA host gene
HPA: Human Protein Atlas
TNM: Tumor node metastasis
ER: Estrogen receptor
Her-2: Human epidermal growth factor receptor 2
HDGF: Hepatoma-derived growth factor
PKM: Pyruvate kinase M1/M2
TNBC: Triple-negative breast cancer
ITGA5: Integrin α5
RAB22A: Ras-related proteins 22a
HOXC8: Homeobox c8
KLF2: Kruppel like factor 2
p21: P21 protein
LC: Laryngeal cancer
MMP2: Matrix metalloproteinase-2
MMP9: Matrix metalloproteinase-9
WEE1: Wee1-like protein kinase
YAP1: Yes-associated protein 1
GC: Gastric cancer
*Hp*: *Helicobacter pylori*
EZH2: Enhancer of Zeste Homolog 2
MED18: Mediator subunit18
CRC: Colorectal cancer
RUNX2: Runt-related transcription factor 2
RCC: Renal cell carcinoma
TOP2A: The expression of topoisomerase IIα
HCC: Hepatocellular carcinoma
OS: Overall survival
BMI1: B lymphoma Mo-MLV insertion region 1
EMT: Epithelial–mesenchymal transition
SMAD3: Sma and Mad Related Family 3
ZEB1: Zinc finger E-box binding homeobox 1
NSCLC: Non-small-cell lung cancer
E2F1: E2F transcription factor 1
TGF-β: Transforming growth factor-β
JAK2: Janus kinase 2
STAT3: Signal transducer and activator of transcription 3
AML: Acute myeloid leukemia
SRGN: Serglycin
DNB: Dynamic Network Biomarkers
CML: Chronic myeloid leukemia
EOC: Epithelial ovarian cancer
OC: Ovarian cancer
FIGO: International federation of gynecology and obstetrics
GSK3β: Glycogen synthase kinase 3 beta
EIF4A3: Eukaryotic translation initiation factor 4A3
PPPM: Parents' Postoperative Pain Measure
SMURF1: Smad Ubiquitin Regulatory Factor 1
GINS2: GINS complex subunit 2
PSMD10: Proteasome 26S subunit non-ATPase 10
PTC: Papillary thyroid carcinoma
OSCC: Oral squamous cell carcinoma
ELAVL1: ELAV-like RNA-binding protein 1
NFYC: Nuclear transcription factor Y subunit gamma
ccRCC: Clear cell renal cell carcinoma
siRNAs: Small interfering RNA
piRNAs: Piwi-interacting RNA
RISC: SiRNA-induced silencing complex
RNAi: RNAi interference
ASOs: Antisense oligonucleotides
